# Physicochemical Characterization and *In Vivo* Evaluation of Amorphous and Partially Crystalline Calcium Phosphate Coatings Fabricated on Ti-6Al-4V Implants by the Plasma Spray Method

**DOI:** 10.1155/2012/603826

**Published:** 2012-08-27

**Authors:** Estevam A. Bonfante, Lukasz Witek, Nick Tovar, Marcelo Suzuki, Charles Marin, Rodrigo Granato, Paulo G. Coelho

**Affiliations:** ^1^Postgraduate Program in Dentistry, UNIGRANRIO University-School of Health Sciences, Rua Professor José de Souza Herdy, 1.160-25 de Agosto 25071-202, Duque de Caxias, RJ, Brazil; ^2^Department of Biomaterials and Biomimetics, New York University, 345E 24th Street Room 813A, New York NY, 10010, USA; ^3^Department of Prosthodontics and Operative Dentistry, Tufts University School of Dental Medicine, One Kneeland Street, Boston, MA, 02111, USA; ^4^Director for Research, Department of Periodontology and Implant Dentistry, New York University College of Dentistry, 345E 24th Street, New York, NY 10010, USA

## Abstract

*Objective*. To characterize the topographic and chemical properties of 2 bioceramic coated plateau root form implant surfaces and evaluate their histomorphometric differences at 6 and 12 weeks *in vivo*. *Methods*. Plasma sprayed hydroxyapatite (PSHA) and amorphous calcium phosphate (ACP) surfaces were characterized by scanning electron microscopy (SEM), interferometry (IFM), X-ray diffraction (XRD), and Fourier transform infrared spectroscopy (FT-IR). Implants were placed in the radius epiphysis, and the right limb of dogs provided implants that remained for 6 weeks, and the left limb provided implants that remained 12 weeks *in vivo*. Thin sections were prepared for bone-to-implant contact (BIC) and bone-area-fraction occupancy (BAFO) measurements (evaluated by Friedman analysis *P* < 0.05). *Results*. Significantly, higher *S_a_* (*P* < 0.03) and *S_q_* (*P* < 0.02) were observed for ACP relative to PSHA. Chemical analysis revealed significantly higher HA, calcium phosphate, and calcium pyrophosphate for the PSHA surface. BIC and BAFO measurements showed no differences between surfaces. Lamellar bone formation in close contact with implant surfaces and within the healing chambers was observed for both groups. *Conclusion*. Given topographical and chemical differences between PSHA and ACP surfaces, bone morphology and histomorphometric evaluated parameters showed that both surfaces were osseoconductive in plateau root form implants.

## 1. Introduction

Most common approaches for dental implant surface modifications involve physically altering the topography or changes in the chemical composition of the surface with the incorporation of inorganic phases [1]. Although topographical changes alone may result in chemistry and physical alterations [2], it has been suggested that improved bone response can be obtained with moderately rough surfaces with *S*_*a*_ values between 1 and 2 *μ*m [3, 4]. Concerning chemical alterations, implant surface incorporation with hydroxyapatite or other calcium phosphate (CaP) compositions has gained significant attention and is amongst the most investigated implant surface modifications [2]. Regardless of engineering surface alteration method, its final aim is to foster the early bone implant healing events allowing prostheses installation and function at earlier time frames than initially proposed [5–7].

Also relevant to the osseointegration healing pathways is the implant macrogeometry [8–10]. Following implantation, implant macrogeometry resulting in intimate contact with bone, a typical scenario in screw root form implants, has shown appositional bone healing along with extensive remodelation occurring before lamellar bone formation [11, 12]. Conversely, the stabilization of the implant device with the tip of the plateaus allowing for formation of healing chambers that are filled with blood clot followed by the formation of woven bone and its replacement with lamellar bone results in a intramembranous healing mode (instead of appositional), commonly observed in plateau root form implants [13–15].

Changes in surface treatment of plateau root form implants aiming to increase texture and chemistry have been described. Compared to a machined surface, qualitative differences in bone formation have been depicted in alumina-blasted/acid-etched (AB/AE), bioceramic deposition on the nanometric scale, and plasma-sprayed calcium phosphate surfaces. Whereas the machined surface has shown bone formation only at the center of the healing chamber, the remaining surfaces presented woven bone formation also in close proximity with the implant surface, suggesting higher osseoconductive properties. Torque to interfacial fracture testing showed significantly higher values for the plasma-sprayed calcium phosphate surface compared to the others [13]. When healing chamber dimensions were addressed in three different surfaces, such as bioactive ceramic electrodeposition, AB/AE, and resorbable blasting media (RBM), surface treatment played a major role in torque to interfacial failure results, where either cylindrical, small or larger healing chambers macrogeometric configurations resulted in significantly higher torque to interfacial fracture for the bioceramic electrodeposition treated surface [11]. Considering that the latter surface presented an overall lower roughness compared to other surfaces, it can be speculated that the surface chemistry including significantly higher amounts of Ca and P (10 at.% and 8 at.%, resp., versus 1 to 3.5 at.% and 1 to 2 at. % for the RBM) likely contributed to the improved bone response.

While investigations concerning implant surface modification performance during early stages of osseointegration have been conducted with plateau root form implants, few studies have addressed the physical and chemical characterization of bioactive ceramic coated surfaces and their effect on osseointegration. This study aimed to characterize plasma sprayed hydroxyapatite (PSHA) and amorphous calcium phosphate (ACP) surfaces with four methods [1] and to histomorphometrically evaluate them at different times *in vivo*.

## 2. Materials and Methods

 The implants used in this study were plateau root form (Ti-6Al-4V) implants with 4.5 mm of diameter and 8.0 mm in length provided by the manufacturer (Bicon LLC, Boston, MA, USA). A total of 36 implants were used and divided in two groups according to surface treatment: plasma-sprayed hydroxyapatite (PSHA) and plasma-sprayed amorphous calcium-phosphate (ACP) (*n* = 18 per group). Six implants per group were used for surface characterization. The manufacturer provided no information regarding the processing parameters of any of the surfaces.

### 2.1. Surface Characterization

 The surface characterization was accomplished with four different methods (*n* = 6 implants per surface). First, scanning electron microscopy (SEM) (Philips XL 30, Eindhoven, The Netherlands) was performed at various magnifications under an acceleration voltage of 15 and 20 kV to observe the different groups' surfaces topography.

The second step was to determine the roughness parameters by optical interferometry (IFM) (Phase View 2.6, Palaiseau, France). Three implants of each surface were evaluated at the flat region of the implant cutting edges (three measurements per implant) and *S*_*a*_ (arithmetic average high deviation) and *S*_*q*_ (root mean square) parameters determined. A filter size of 100 *μ*m × 100 *μ*m was utilized. Following data normality verification, statistical analysis at 95% level of significance was performed by one-way ANOVA.

A XRD (X'Pert X-Ray diffractometer, Philips, Andover, MA, USA) was used to determine the crystalline phases present within the PSHA and ACP. For this purpose, the coating was scrapped off 6 implants per group for sufficient material for powder diffraction. Three different spectra from each implants coating were obtained. The diffractometer, used a curved crystal monochromator, operating at 45 mA and 45 kV, and scanned in the 2*θ* with a range from 20 to 40°, with a step size of 0.02° at 3 seconds per step.

Rietveld refinement analysis used the data collected from Philips X'Pert X-Ray diffractometer, which subsequently converted data to “.xy” format. The raw data was then input into, Material Analysis Using Diffraction (MAUD) software for quantitative analysis. Rietveld analysis utilized the samples from previous XRD spectra (i.e., peak, heights, widths, and positions). This refinement method was used to quantify the percentage of each phase (HA, *β*-TCP, and other commonly observed phases in calcium- and phosphate-based materials such as calcium oxide, (CaO) present in the different coatings. A least square fit approach was utilized to measured scans until replicating a theoretical scan (based on the structure in Inorganic Crystal Structure Data Base-ICSD). The background, cell parameters, preferred orientation, peak asymmetry, atomic positions, site occupancy factors, and global vibrational parameters were refined. The calculated and observed patterns were plotted by least squares method until a minimum was reached. Five iterations were utilized and the integrated intensities, and the peaks heights were related to a scale factor. The fraction of each phase was determined by

(1) Wi=Si(ZMV)i∑[Sj(ZMVj)],

where, *W*_*i*_ is the weight fraction of the phase, *S* is the scale factor, *Z* is the number of formulas per unit cell, *M* is the mass of the formula unit, *V* the unit cell volume, and *i* and *j* are the phase under analysis and *j* all phases in the system.

Finally, FT-IR (NicoletMagna IR 550 Spectrometer Series II) analyses were made for the different coatings. Pellets were fabricated by mixing 1 milligram of the desired coating material with 250 milligrams KBr (IR grade, Thermo Scientific) and using a hydraulic press (CrushIR, Pike Technologies, Watertown, WI, USA). A range of 4000 to 400 cm^−1^ was scanned. 

### 2.2. Animal Model and Surgical Procedure

 The *in vivo *evaluation comprised 6 adult male beagles of approximately 1.5 years old. Approval from the Ethics Committee for Animal Research at the École Nationale Vétérinaire d'Alfort (Maisons-Alfort, Val-de-Marne, France). The beagles remained in the facility for two weeks prior to the surgical procedures.

For surgery, three drugs were administered until general anesthesia achievement by intramuscular injection. The drugs were atropine sulfate (0.044 mg/kg), xylazine chlorate (8 mg/kg), and Ketamine chlorate (15 mg/kg). The implantation site was the radius epiphysis, and the right limb of each animal provided implants that remained for 6 weeks *in vivo*, and the left limb provided implants that remained 12 weeks *in vivo*.

For implant placement, the surgical site was shaved with a razor blade and was followed by application of antiseptic iodine solution. An incision of ~5 cm through the skin and periosteum was performed, and the periosteum was elevated for bone exposure.

 Sequential drills were utilized following the manufacturer's recommendation under abundant saline irrigation at 1,200 rpm. The two-implant groups were alternately placed from proximal to distal at distances of 1 cm from each other along the central region of the bone. In order to minimize bias in histomorphometric measurements, the starting implant surface was also alternated between dogs. This approach enabled the symmetrical evaluation of the 2 surfaces per animal limb, site, and time *in vivo.*

After placement the site was sutured in layers with vicryl 4-0 (Ethicon Johnson, Miami, FL, USA) for periosteum and nylon 4-0 (Ethicon Johnson, Miami, FL, USA) for skin was performed. The animals stayed in animal care facility and received antibiotic (Benzyl Penicilin Benzatine 20.000 UI/Kg) and anti-inflammatory (Ketoprofen 1% 1 mL/5 Kg) medication to control the pain and infection. Euthanasia by anesthesia overdose was performed after 6 and 12 weeks, the limbs were retrieved by sharp dissection.

The implants in bone were then referred to histomorphometric analysis. The implants in bone were reduced to blocks and were then immersed in 10% buffered formalin solution for 24 h. The blocks were then washed in running water for 24 h, and gradually dehydrated in a series of alcohol solutions ranging from 70 to 100% ethanol. Following dehydration, the samples were embedded in a methacrylate-based resin (Technovit 9100, Heraeus Kulzer GmbH, Wehrheim, Germany) according to the manufacturer's instructions. The blocks were then cut into slices (~300 *μ*m thickness) aiming the center of the implant along its long axis with a precision diamond saw (Isomet 2000, Buehler Ltd., Lake Bluff, USA), glued to acrylic plates with an acrylate-based cement, and a 24 h setting time was allowed prior to grinding and polishing. The sections were then reduced to a final thickness of ~50 *μ*m by means of a series of SiC abrasive papers (400, 600, 800, 1200, and 2400) (Buehler Ltd., Lake Bluff, IL, USA) in a grinding/polishing machine (Metaserv 3000, Buehler Ltd., Lake Bluff, USA) under water irrigation [16]. The sections were then toluidine blue stained and referred to optical microscopy for histomorphologic evaluation.

The bone-to-implant contact (BIC) was determined at 50x–200x magnification (Leica DM2500M, Leica Microsystems GmbH, Wetzlar, Germany) by means of computer software (Leica Application Suite, Leica Microsystems GmbH, Wetzlar, Germany). The regions of bone-to-implant contact along the implant perimeter were subtracted from the total implant perimeter, and calculations were performed to determine the BIC. The bone area fraction occupied (BAFO) between threads in trabecular bone regions was determined at 100x magnification (Leica DM2500M, Leica Microsystems GmbH, Wetzlar, Germany) by means of computer software (Leica Application Suite, Leica Microsystems GmbH, Wetzlar, Germany). The areas occupied by bone were subtracted from the total area between threads, and calculations were performed to determine the BAFO (reported in percentage values of bone area fraction occupied) [9]. Statistical evaluation of BAFO and BIC was performed by Friedman analysis. Statistical significance was set to 95% level. For the IFM and Rietveld quantification, *t*-tests at 95% level of significance were employed.

## 3. Results

**Figure 1:**
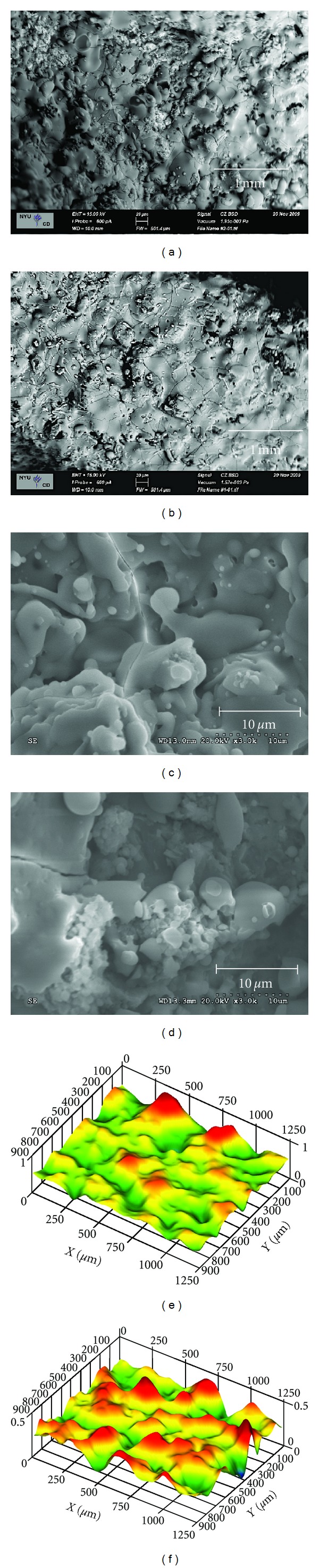
SEM intermediate and high magnification micrographs for the ACP ((a) and (c)) and PSHA ((b) and (d)) surfaces. Representative 100 *μ*m × 100 *μ*m IFM three-dimensional reconstructions for PSHA (e) and ACP (f).

**Figure 2:**
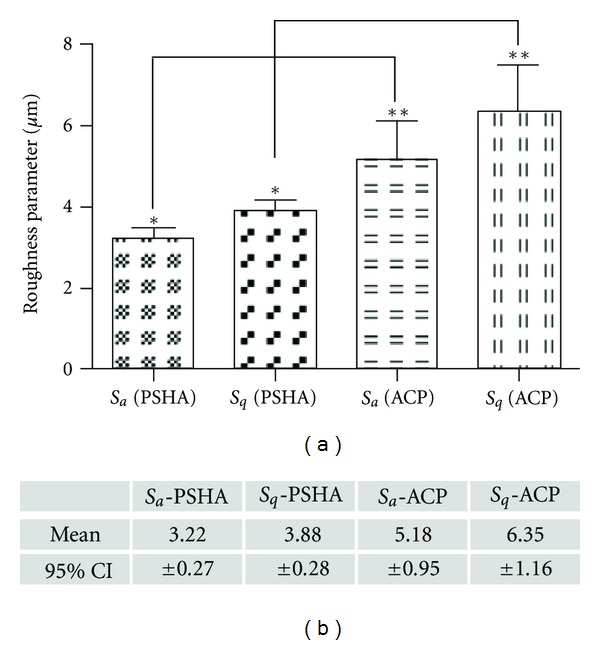
Micrometer *S*_*a*_ and *S*_*q*_ measurements resulted in significant differences for *S*_*a*_ (*P* < 0.03) and *S*_*q*_ (*P* < 0.02) between PSHA and ACP, with the ACP surface presenting higher values, as shown in the graphic and table.

 The PSHA and ACP implant surfaces' SEM images, as well as their representative 100 *μ*m × 100 *μ*m IFM three-dimensional reconstructions are presented in Figures 1 and 2, respectively. The surface texture observed at intermediate and high magnification levels (Figure 1), as well as the IFM reconstruction (Figure 2) revealed minimal morphologic differences between the two groups. The individual micrometer *S*_*a*_ and *S*_*q*_ length IFM measurements (Figure 2) resulted in significant differences for *S*_*a*_ (*P* < 0.03) and *S*_*q*_ (*P* < 0.02) between PSHA and ACP, with the ACP surface presenting higher values.

**Figure 3:**
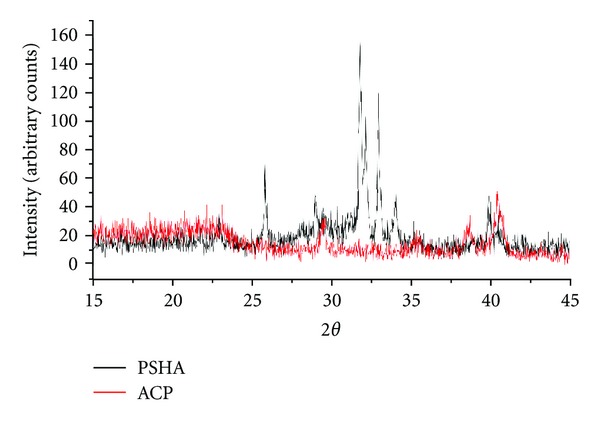
XRD spectra for the two surface groups for crystalline verification.

**Figure 4:**
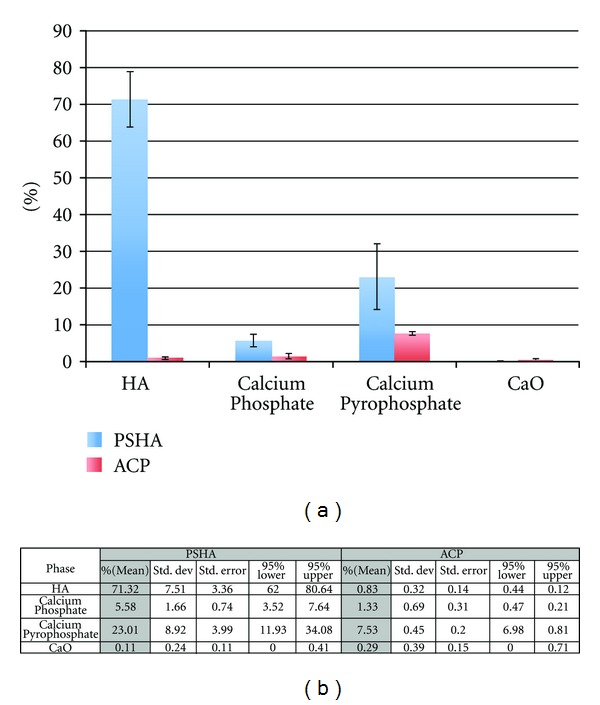
(a) Rietveld analysis using MAUD program for crystalline phase quantification of both PSHA and ACP surfaces. (b) Shows the phase fraction mean and 95% confidence intervals.

 XRD spectra were collected for the two surface groups (Figure 3) for crystalline verification. The surface coating materials were then subjected to the Rietveld analysis using MAUD program for crystalline phase quantification. The phase fraction mean and 95% confidence intervals are presented in Figure 4.

**Figure 5:**
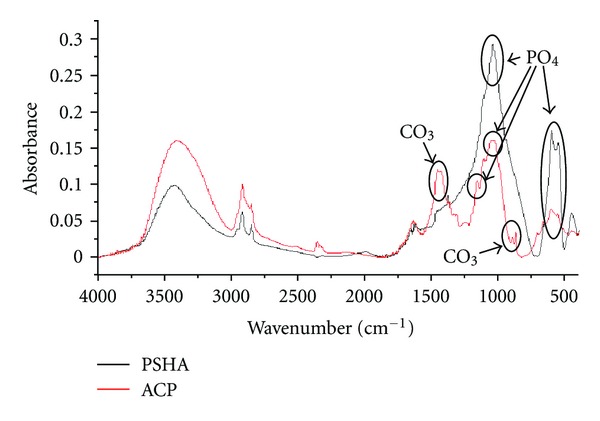
Stacked FT-IR spectrum showing the chemical groups and respective absorbance for PSHA and ACP. Carbonate groups were only observed in the ACP coating.

FT-IR was executed to explore the chemical groups molecular vibration. Phosphate group (PO_4_) peaks of interest were located around wavenumbers from 1100 to 1040 cm^−1^ as well as double peaks present in the ~600 to 560 cm^−1^ range, while peaks positioned in the 850 to 800 cm^−1^ areas were indicative of a carbonate group (CO_3_). A stacked FT-IR spectrum comparing PSHA and ACP chemical groups and respective absorbance's is displayed in Figure 5. A key observation between the two implant surfaces is the presence of the carbonate groups (labeled in Figure 5) in the ACP coating in comparison to the lack there of in PSHA coating.

The surgical procedures and followup exhibited no complications with respect to procedural conditions, postoperative infection, or any other clinical concerns.

**Figure 6:**
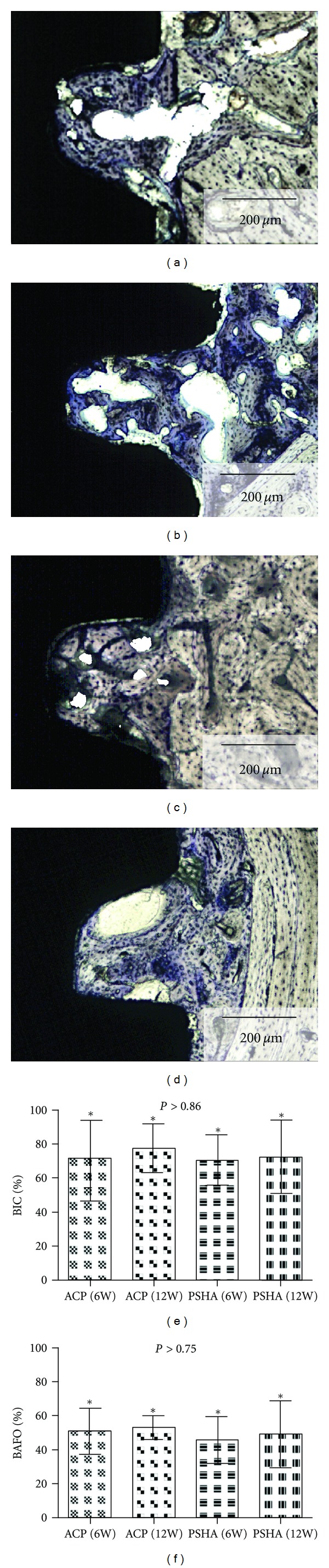
Intimate contact between bone and implant surfaces was observed at 6 weeks (a) PSHA group and (c) ACP group. Woven bone formation at the healing chamber region, where no contact between bone and implant existed after device implantation, is depicted as well as its formation in close proximity to the implant surface. At 12 weeks, lamellar bone replacing woven bone was observed for both (b) PSHA and (d) ACP surfaces. The histomorphometric evaluation exhibited no significant differences between the two surfaces at both times *in vivo* (6 and 12 weeks) for (e) BIC (*P* > 0.86), and (f) BAFO (*P* > 0.75).

 Evaluation of the toluidine blue stained thin sections qualitatively indicated intimate contact between bone and the two implant surfaces at 6 weeks. Woven bone formation at the healing chamber region, where no contact between bone and implant existed after device implantation, was observed (Figure 6). At 12 weeks, lamellar bone replacing woven bone was observed at the healing chambers for both implant surfaces. The histomorphometric evaluation exhibited no significant differences between the two surfaces at both times *in vivo* (6 and 12 weeks) for BIC (*P* > 0.86) and BAFO (*P* > 0.75).

## 4. Discussion

Because of its high survival rates over the long-term, implant dentistry is considered one of the most successful rehabilitation modalities in the medical field [17–20]. Although implant surface treatment has been pointed as a key factor for clinical success in the past [21], long-term evidence to support the indication of one surface relative to another is lacking. However, considering the impact of missing teeth on oral health related quality of life [22], the possibility of implant loading at earlier time frames has been the driving force in surface engineering design with significant benefit to patients. Furthermore, evidence from *in vitro* and *in vivo* investigations strongly suggests that more rapid bone formation can be achieved with treated surfaces relative to machined ones [3–5, 23, 24]. The current issue with understanding improved bone to implant interaction is the dearth of in-depth surface properties characterization on both topographical and composition levels [25].

 It has been previously suggested that insufficient surface characterization hinders the understanding of the influence of surface modifications on early osseointegration events [2, 3, 26]. For this reason, PSHA and ACP treated surfaces were characterized with 4 recommended analytical tools [1]. Both *S*_*a*_ and *S*_*q*_roughness parameter values fell in the rough range classification [6] and were significantly higher for the ACP surface compared to the PSHA. As per the Rietveld analysis, the mean values for HA, calcium phosphate, and calcium pyrophosphate were substantially higher than for the ACP surface, yet with no differences in both BIC and BAFO histomorphometric parameters. Therefore, neither topographical nor chemical differences between evaluated surfaces affected the evaluated parameters.

The specific topographical and chemical characteristics of a bioactive ceramic surface tailored for improved bone response are unknown. It has been shown that the amounts of CaP is critical to elicit improved bone/implant biomechanical response, regardless of surface topography [27, 28]. Blasting with ceramic particles followed by a nonwashing procedure aimed to increase the CaP amounts has shown to significantly improve the torque to interfacial fracture of a RBM surface compared to a AB/AE with higher *S*_*a*_ and *S*_*q*_, although BIC and BAFO morphometric measurements were not significantly different [29]. Although significantly higher CaP was observed for the PSHA relative to the ACP in the present study, the evaluated static morphometric parameters did not detect any differences between surfaces. Future biomechanical testing is warranted to address potential differences between surfaces.

Although PSHA coated implant surfaces have shown substantial improvements in bone to bioceramic bonding and BIC [30–32], issues such as nonuniform degradation over the long term, compromised coating, and bone-coating interface mechanical properties, as well as adhesive failures (bulk metal, metal oxide, and bioceramic coating) [33–35] lead to the development of several techniques to incorporate bioceramics onto surfaces [5]. Retrieval analysis of more recent PSHA coated surfaces on plateau root form implants was able to show the temporal behavior from short (2 months) to long term (13 years) in humans. Regardless of time in function, lamellar bone was observed in close contact with the PSHA coating with average BIC of 65% (similar to the present findings), and Haversian-like osteonic morphology between plateaus [36]. Similar bone morphology has been observed for AB/AE coated implants retrieved after 8 to 13 years in function and also removed due to prosthetic reasons. Mean BIC value was 62.2% and harvesian-like microstructure running perpendicular to and along the implant's long axis was observed at cortical and trabecular bone [37]. Although morphometric and morphological differences could not be detected between the AB/AE and PSHA surfaces in the retrieval studies, future studies addressing the temporal changes in biomechanical properties, such as bone modulus of elasticity and hardness at the implant/bone interface are warranted. Finally, recently described techniques for calcium phosphate coatings such as template-assisted electrohydrodynamic atomization have shown to produce a tailored topography at both the micro and nanoscale [38], as well as electrohydrodynamic print-patterning that generates ordered topographies with controlled porosity and bioactivity [39], and they are both promising and also warrant future *in vivo* and clinical studies.
